# Study on the influence mechanism of environmental perception on physical health status—with lifestyle as the mediating variable

**DOI:** 10.3389/fpubh.2025.1644565

**Published:** 2026-01-12

**Authors:** Rong Lin, Xianghui Zhou

**Affiliations:** 1 School of Law, Anhui Normal University, Wuhu, China; 2 School of Mathematics and Statistics, Anhui Normal University, Wuhu, China

**Keywords:** environmental perception, health, lifestyle, mediating effect, multinomial Logit model

## Abstract

Residents’ environmental perception affects lifestyle choices, which in turn shape health status. Using 2,006 valid samples from the Chinese General Social Survey (CGSS2021), this study employed the multinomial Logit model and mediating effect model to explore the mechanism among environmental perception, lifestyle, and health. Key findings: (1) Residents are optimistic about macro environmental risks (e.g., air/water pollution) but sensitive to specific risks (e.g., vehicle exhaust); (2) Environmental perceptions (e.g., extreme weather, food safety concerns) positively impact health, while air pollution perception shows no direct promoting effect; (3) Unhealthy lifestyles (insufficient physical activity, irregular sleep) harm health, while adequate sleep and leisure/relaxation (especially frequent relaxation) benefit health; (4) Lifestyle plays a key mediating role: positive environmental perception improves health by enhancing social fairness and promoting social activities, while heightened environmental risk perception inhibits regular exercise, triggering “environmental pressure-behavioral disorder-health deterioration.” Innovatively, the study clarifies the chained mechanism of “environmental perception-lifestyle-health” via combined models. Practically, it proposes a health promotion system integrating environmental governance optimization and behavioral intervention, to facilitate a “cognition-behavior-health” virtuous cycle.

## 1 Introduction

As global health awareness continues to grow, it’s clear that individual health is shaped by multiple interconnected factors. The theory of social determinants of health reveals that health disparities aren’t just about biological differences—they are largely driven by complex social mechanisms (1). Non-medical factors like living environment, economic status, and social support play a decisive role in determining health outcomes (2, 3). For instance, people living in areas with severe air pollution and poor sanitation infrastructure suffer from higher rates of respiratory diseases compared to those in ecologically balanced neighborhoods (4). Moreover, individuals with lower socioeconomic status face a dual challenge: they struggle to access quality healthcare and nutritious food, while chronic stress makes them more vulnerable to mental health issues. These interconnected social factors collectively create distinct health disparity patterns across different social groups. A telling example comes from Hu’s et al. (5) study using China Health and Retirement Longitudinal Study data (2011–2018) and PM2.5 satellite monitoring, which revealed significant urban–rural differences in how air pollution affects middle-aged and older adult. Therefore, understanding the relationship between environment and individual health requires careful examination of how various social factors work together as a whole.

It is evident that the living environment directly shapes residents’ environmental perceptions, which in turn influence physical health through an interconnected “physiological-psychological-behavioral” process. The direct pathway demonstrates the mind’s immediate control over bodily responses (6, 7), while the indirect pathway reveals how behavioral patterns serve as key bridges linking environmental impact to health outcomes (8, 9). This establishes “environmental perception and physical health” as a critical research theme. For instance, Russ (10) investigated the potential effects of environmental perception on human health, while Adanu et al. (11) demonstrated significant correlations between perceived environmental hazards and health risks. Notably, negative environmental perceptions may lead residents to reduce outdoor social activities and increasingly adopt sedentary indoor entertainment (e.g., prolonged television viewing), thereby exacerbating loneliness and physical inactivity—ultimately compromising health. This dynamic aligns with Bronfenbrenner’s socio-ecological model (12), which posits that health behaviors emerge from continuous interactions between individuals and multi-layered environmental systems, including living spaces and community contexts. Thus, environmental perceptions—rooted in societal evolution—directly and indirectly shape physical health, creating a fluid interplay that warrants continued scholarly exploration.

It is note that Rogers’ Protection Motivation Theory (1975) further posits that environmental perception directly influences an individual’s willingness to adopt protective behaviors. Higher risk perception strengthens motivation to act, whereas lower awareness weakens protective motivation (13). Consequently, choosing healthy lifestyles—such as maintaining regular physical activity, consistent sleep patterns, adequate relaxation, balanced fruit intake, and social engagement—can foster positive interactions between individuals and their environment. These constructive lifestyle practices enhance residents’ adaptability to their surroundings and reduce environment-related health risks (14). For instance, Makeen et al. (15) conducted in-depth analyses of the complex interrelationships among different lifestyle factors, revealing diverse risk perception patterns and varying health outcomes associated with different behavioral choices (16). This demonstrates that lifestyle serves as an essential pathway through which environmental perception affects physical health. Therefore, when examining the relationship between environmental perception and health outcomes, lifestyle must be considered an indispensable mediating variable. Regarding research approaches and perspectives, existing studies often examine environmental perception or lifestyle in isolation when exploring their connections to physical health (17–19). However, quantitative investigation into the underlying mechanism of how environmental perception influences health through lifestyle remains insufficient—representing a key research motivation for this study.

The impact of lifestyle on health and its quantitative assessment require further research. As a multidimensional variable encompassing perceptions of the physical, social, and psychological environments, environmental perception systematically influences residents’ lifestyle choices through their subjective interpretation of environmental factors, ultimately manifesting in health outcomes. We know that individual healthy lifestyle choices are affected by numerous factors, such as education level, health awareness, dietary habits, and economic income. Relevant studies indicate that individuals with higher health awareness are more inclined to consciously cultivate healthy lifestyles; similarly, those with higher education levels tend to adopt more scientifically sound health behaviors due to their deeper understanding of health knowledge (20, 21). Although existing research has extensively explored the impact of lifestyle on physical health, most studies remain theoretical or focus primarily on statistical analysis (22). There is a lack of theoretical interpretation supported by multi-case comparisons or empirical testing, and the robustness of their conclusions still needs verification. Establishing a “environmental perception—lifestyle—physical health” causal chain with lifestyle as the mediating variable, and analyzing how environmental perception drives lifestyle choices (23) to reveal the internal mechanism between environment and health—this constitutes the second research motivation of this paper.

This study focuses on the domain of physical health, employing lifestyle as a mediating variable to examine its dynamic mechanism within the relationship between environmental perception and physical health. Guided by the “environmental perception → lifestyle choices → health status” analytical framework, we utilize baseline regression and multinomial Logit models for quantitative analysis, aiming to unveil the underlying pathways and transmission effects among these variables. Based on this foundation, we propose the following research hypotheses to advance subsequent investigation.

*H1*: Environmental perceptions exert a significant and differentiated impact on lifestyles. Specifically, the perception of environmental factors related to food and residential safety demonstrates a positive predictive effect on the adoption of healthy lifestyle behaviors.

*H2*: On weekdays, opting for adequate sleep and relaxation is the preferred choice for residents to maintain their health.

*H3*: Environmental perception exerts differential effects on residents’ physical health, depending on its specific level.

*H4*: Lifestyle plays a mediating role in the relationship between environmental perception and physical health, whereby individuals’ lifestyle choices lead to varied health outcomes.

## 2 Data source, reconstruction and specification

The data used in this study is sourced from the database of the Chinese General Social Survey (CGSS2021) in 2021.^1^ This survey adopted a multi-stage stratified sampling method, taking a total of 2,801 district and county units in 22 provinces, 4 autonomous regions (excluding the Tibet Autonomous Region), and 4 directly-administered municipalities (excluding Hong Kong, Macau and Taiwan) in China as the primary sampling units (PSUs). In this paper, the core independent variables of these models are environmental perception level,^2^ the mediating variable is lifestyle,^3^ and the explained variables are physical health status.^4^

After data cleaning, by removing the missing values and invalid records in the research indicators, a total of 2,006 valid samples were finally obtained. Among them, males accounted for 45.8% and females accounted for 54.2%. To ensure the consistency of variable measurement standards during the model construction process, all variables were quantitatively coded using a five-point scale, to achieve the standardization of data dimensions. The specific operations of data reconstruction and standardized assignment are as follows (Other variables adopt the original 5-point Likert scale data structure):

1. Variables of environmental hazard perception.

For the three variables of the perception of the hazards of vehicle exhaust pollution (VehExh), the perception of the hazards of pesticides and fertilizers (PesFer), and the perception of the hazards of genetically modified crops (GenCr), the corresponding data of the “unable to choose” option in the original questionnaire were deleted, and the other options were re-assigned values as follows: 1 = no hazard at all, 2 = not very harmful, 3 = somewhat harmful, 4 = very harmful, 5 = extremely harmful to the environment, so as to achieve the quantitative representation of the degree of hazard.

1. Variables of food safety perception.

For the variable of the perception of food safety in the residential area (FoSaf), the value of 7 assigned to the option “There is no such problem” in the original questionnaire was adjusted to the category of “not serious.” At the same time, the invalid data of “unable to answer” were removed to ensure the validity of the data.

1. Variables of living environment perception.

For the three perception variables of the suitability for physical exercise within 1 km of the place of residence (LivEnv), the availability of fresh fruits and vegetables (VegFru), and the degree of approval of public facilities (libraries, parks, etc.) (PubFac), the values were reassigned as follows: 1 = completely disagree, 2 = disagree, 3 = neither agree nor disagree, 4 = agree, 5 = completely agree, so as to achieve the standardization of attitude measurement.

1. Variables of lifestyle.

For the physical activity that makes the breath faster than usual per week (Lifestyle_1_), the values were reassigned according to the activity duration: 1 = less than 30 min, 2 = 30–60 min, 3 = 2–3 h, 4 = 4–5 h, 5 = 6 h and above.

For the regularity of the usual work schedule arrangement (Lifestyle_3_), it was reassigned according to the work pattern as follows: 1 = other work schedules, 2 = work schedules change frequently, 3 = shift rotation system, 4 = regular night shift, 5 = regular day shift.

For the actual sleep duration on general working days (Lifestyle_5_), it was reassigned according to the degree of health as: (1) (<4 h, short sleep), (2) (4–6 h, relatively low sleep), (3) (6–8 h, moderate sleep), (4) (8–10 h, relatively high sleep), (5) (>10 h, long sleep).

For the frequency of social and entertainment activities with other friends (Lifestyle_7_), it was reassigned according to the frequency of activities as: 1 = once a year or less, 2 = several times a year, 3 = once or several times a month, 4 = 1–2 times a week, 5 = almost every day.

1. Treatment of dependent variables.

The “current physical health status” (Health) was set as the dependent variable of the study, and its values were reassigned as: 1 = poor, 2 = average, 3 = good, 4 = very good, 5 = excellent, so as to establish a unified quantitative standard for the subsequent analysis of the influencing factors of health.

The core variable explanations and some specifications are presented in Table 1. In particular, the present research focuses on investigating the impact of environmental perception levels and lifestyle choices on residents’ physical health. Accordingly, the independent variables of the proposed model are primarily anchored in environmental perception variables, which serve as the core explanatory variables. Nevertheless, residents’ social attribute characteristics constitute non-negligible factors. To validate the robustness of the established model, the three characteristic variables—i.e., “Gender,” “Education Level,” and “Economic Income”—were incorporated into the correspondingly constructed model in the experimental results and analysis section. Subsequent to recalculating the model parameters, a comparative analysis was conducted against the previously derived results. This analytical procedure aims to elucidate the influence of residents’ social attributes (among the independent variables) on both the mediating variable (lifestyle) and the explained variable (physical health status).

**Table 1:** Variable explanations and specification.

Variable	Variable explanations	Mode	Mean	SD
AirP	Perception of air pollution; 1 = no impact at all; 2 = a little impact; 3 = some impact; 4 = a great deal of impact; 5 = a very great impact	1	1.9426	0.9745
WaterP	Perception of water pollution; 1 = no impact at all; 2 = a little impact; 3 = some impact; 4 = a great deal of impact; 5 = a very great impact	1	1.8833	0.9999
ExtWea	Perception of the impact of extreme weather; 1 = no impact at all; 2 = a little impact; 3 = some impact; 4 = a great deal of impact; 5 = a very great impact	1	2.0104	1.0693
VehExh	Perception of the harm of vehicle exhaust pollution; 1 = no harm at all; 2 = not very harmful; 3 = somewhat harmful; 4 = very harmful; 5 = extremely harmful to the environment	3	3.6076	0.8326
PesFer	Perception of the harm of pesticides and fertilizers; 1 = no harm at all; 2 = not very harmful; 3 = somewhat harmful; 4 = very harmful; 5 = extremely harmful to the environment	3	3.3983	0.9150
GenCr	Perception of the harm of genetically modified crops; 1 = no harm at all; 2 = not very harmful; 3 = somewhat harmful; 4 = very harmful; 5 = extremely harmful to the environment	3	3.1071	0.8603
FoSaf	Perception of food safety in the residential area; 1 = not serious; 2 = not very serious; 3 = moderate; 4 = relatively serious; 5 = very serious	3	4.1096	1.7932
LivEnv	Perception of the suitability for physical exercise within 1 km of the place of residence; 1 = completely disagree; 2 = disagree; 3 = neither agree nor disagree; 4 = agree; 5 = completely agree	4	3.8315	1.0143
VegFru	Perception of the availability of fresh vegetables and fruits in the place of residence; 1 = completely disagree; 2 = disagree; 3 = neither agree nor disagree; 4 = agree; 5 = completely agree	4	4.1306	0.8076
PubFac	Perception of the identification with public facilities (such as libraries, parks, etc.) within 1 km of the place of residence; 1 = completely disagree; 2 = disagree; 3 = neither agree nor disagree; 4 = agree; 5 = completely agree	4	3.2637	1.2381
Lifestyle_1_	The time spent on physical activities that make the breath faster than usual each week; 1 = less than 30 min; 2 = 30 to 60 min; 3 = 2 to 3 h; 4 = 4 to 5 h; 5 = 6 h and above	1	2.1979	1.4611
Lifestyle_2_	The frequency of engaging in physical exercise that makes you sweat or breathe faster for at least 20 min; 1 = never; 2 = once a month or less; 3 = several times a month; 4 = several times a week; 5 = every day	1	2.8883	1.5909
Lifestyle_3_	Regularity of work schedule on weekdays; 1 = other work schedules; 2 = work schedule often changes; 3 = rotational days off system; 4 = regular night shift; 5 = regular day shift	1	1.7696	1.2577
Lifestyle_4_	Perception of social fairness; 1 = completely unfair; 2 = relatively unfair; 3 = it’s hard to say it’s fair, but neither can it be said to be unfair; 4 = relatively fair; 5 = completely fair	4	3.3808	0.9721
Lifestyle_5_	Actual sleep duration on typical workdays; 1 = short sleep level; 2 = lower sleep level; 3 = moderate sleep level; 4 = higher sleep level; 5 = long sleep level	4	3.5832	1.0021
Lifestyle_6_	Whether often taking rest and relaxation in spare time; 1 = never; 2 = seldom; 3 = sometimes; 4 = often; 5 = very frequently	4	3.5583	0.9376
Lifestyle_7_	The frequency of social and recreational activities with friends (such as visiting each other, having meals together, playing cards, etc.); 1 = once a year or less or never; 2 = several times a year; 3 = once or several times a month; 4 = one to two times a week; 5 = almost every day	3	2.5957	1.2643
Physical Health	Current physical health status; 1 = poor; 2 = moderate; 3 = good; 4 = very good; 5 = excellent	4	4.0583	0.6378

## 3 The framework and model construction

### 3.1 Research framework

**Figure 1:**
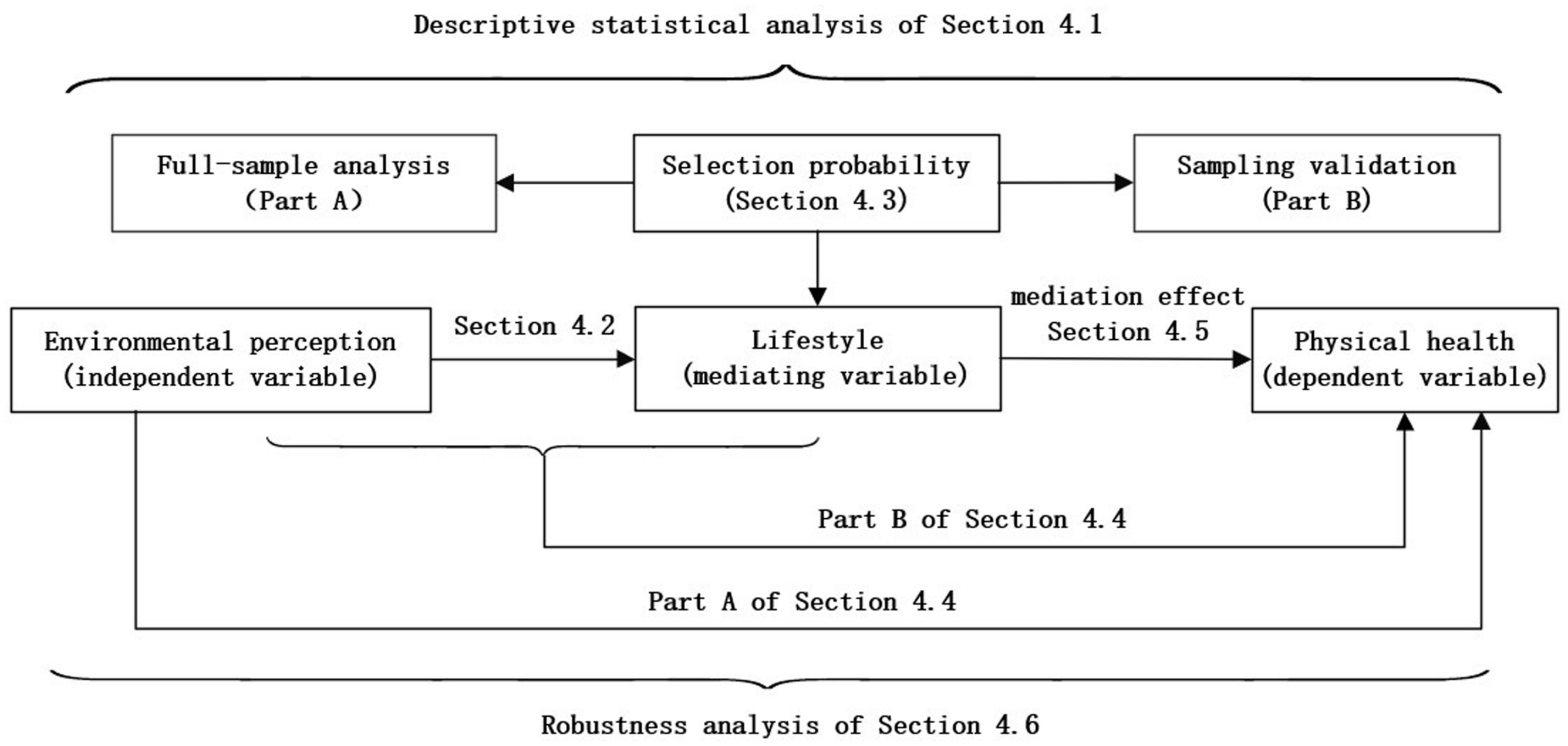
Research framework.

We first provide a statistical description of all selected sample data in Section 4.1, which serves as an initial data exploration. In Section 4.2, we analyze the impact effect of environmental perception (independent variable) on lifestyle (mediating variable) based on Model 1. In Section 4.3, focusing on “lifestyle” and based on Models 2, 3, we use multinomial Logit models to analyze the issue of residents’ lifestyle selection probability from both overall data and partial sampling data. In Section 4.4, we divide the analysis into two parts based on Models 4, 5: the first part examines the impact effect of environmental perception on physical health status (dependent variable); the second part analyzes the impact effects of environmental perception and lifestyle on physical health status after incorporating the mediating variable (lifestyle). In Section 4.5, the analysis mainly focuses on the direct mediating effect and transmission mechanism of the lifestyle on the physical health status. In Section 4.6, to verify the robustness and validity of the results obtained from Models 1, 4, and 5, we incorporate social attribute variables—namely “gender,” “education level,” and “economic income”—into the independent variables of the models respectively, then conduct regression calculations again to test the validity of Models 1, 4, 5. The research framework of this paper are presented in Figure 1.

### 3.2 Research models

In this section, we regress the environmental perception variables on the lifestyle variables, while the coefficients of this regression model are used to calculate the multinomial Logit model. The aim is to obtain the probability of residents’ lifestyle choices under the influence of environmental perception. We then conduct a regression analysis of the environmental perception variables on residents’ physical health variables, and subsequently incorporate the lifestyle to derive a mediating regression model. This enables a closed-loop analysis that captures the effects of “environmental perception” on “lifestyle” and “lifestyle” on “physical health.”

#### 3.2.1 The model of environmental perception on lifestyles

The regression model of mediating effect based on environmental perception is set as follows:


$$\begin{array}{l} \text{Lifestyl}e_{j}=\alpha_{0j}+\alpha_{1j}\text{AirP}+\alpha_{2j}\text{WaterP}+\alpha_{3j}\text{ExtWea}+\\ \alpha_{4j}\text{VehExh}+\alpha_{5j}\text{PesFer}+\alpha_{6j}\text{GenCr}+\\ \alpha_{7j}\text{FoSaf}+\alpha_{8j}\text{LivEnv}+\alpha_{9j}\text{VegFru}+\\ \alpha_{10j}\text{PubFac}+\varepsilon_{j}(j=1,2,\cdots ,7) \end{array}$$ (1)

where AirP,WaterP,ExtWea,VehExh,PesFer,GenCr,FoSaf,LivEnv,VegFru and PubFac are the environmental perception variables, which are also the basic explanatory variables of the model; $\text{Lifestyl}e_{j}\;\;(j=1,2,\ldots ,7)$ are lifestyle choice variables, which are also the mediating variables in this paper, and the interpretations are presented in Table 1; $\alpha_{0j},\alpha_{1j},\cdots ,\alpha_{10j}$ are the parameters of the above model.

#### 3.2.2 Choice probability of lifestyles

This study focuses on the impact of residents’ lifestyle choices. The multinomial Logit model (24, 25) is applied to quantitatively analyze the probability of residents’ choices among different lifestyle options. This study uses a precise statistical model to examine how people’s perception of their environment shapes their lifestyle choices and, ultimately, their health. The model calculates the probability of an individual making a specific choice using the following formula:


$$P(Y_{j}=F_{j})=\frac{e^{V_{\mathrm{ij}}}}{\sum_{j=1}^{m}e^{V_{\mathrm{ij}}}}=\frac{e^{\alpha_{0j}+\alpha_{1j}X_{1}+\alpha_{2j}X_{2}+\cdots +\alpha_{\mathrm{pj}}X_{p}}}{\sum_{j=1}^{m}e^{\alpha_{0j}+\alpha_{1j}X_{1}+\alpha_{2j}X_{2}+\cdots +\alpha_{\mathrm{pj}}X_{p}}}$$

If the reference category is set as $Y_{0}=F_{1}$ , then the selection probability of the reference category is as follows:


$$P(Y_{0}=F_{1})=\frac{e^{0}}{e^{0}+\sum_{j=2}^{m}e^{\alpha_{0j}+\alpha_{1j}X_{1}+\alpha_{2j}X_{2}+\cdots +\alpha_{\mathrm{pj}}X_{p}}}.$$ (2)

The probability that an individual selects other categories is


$$P(Y_{j}=F_{j}|Y_{0}=F_{1})=\frac{e^{\alpha_{0j}+\alpha_{1j}X_{1}+\alpha_{2j}X_{2}+\cdots +\alpha_{\mathrm{pj}}X_{p}}}{e^{0}+\sum_{j=2}^{m}e^{\alpha_{0j}+\alpha_{1j}X_{1}+\alpha_{2j}X_{2}+\cdots +\alpha_{\mathrm{pj}}X_{p}}}.$$ (3)

Among them, $Y_{j}$ represents the choice variable of the model, j represents the corresponding category ordinal number, and j∈[1,2,…,m] ; i represents the individual making the choice; $X_{1},X_{2},\cdots ,X_{p}$ represent the explanatory variables of the model; $V_{\mathrm{ij}}$ represents the utility function for individual i to select a certain category $F_{j}$ ; $\alpha_{0j},\alpha_{1j},\cdots ,\alpha_{\mathrm{pj}}$ are the model coefficients of the utility function.

#### 3.2.3 Basic regression model of environmental perception and health

Ten environmental perception variables are employed as the independent variables in the basic regression model, meanwhile physical health is designated as the explained variable of the model. The specification of this model is formulated as follows:


$$\begin{array}{l} \text{Health}=\lambda_{0}+\lambda_{1}\text{AirP}+\lambda_{2}\text{WaterP}+\lambda_{3}\text{ExtWea}+\\ \lambda_{4}\text{VehExh}+\lambda_{5}\text{PesFer}+\lambda_{6}\text{GenCr}+\\ \lambda_{7}\text{FoSaf}+\lambda_{8}\text{LivEnv}+\lambda_{9}\text{VegFru}+\\ \lambda_{10}\text{PubFac}+\varepsilon , \end{array}$$ (4)

where AirP,WaterP,ExtWea,VehExh,PesFer,GenCr,FoSaf,LivEnv,VegFru and PubFac are the environmental perception variables, which are also the basic explanatory variables of the model. Health is the dependent variable of the model, and the interpretation of its variable is shown in Table 1. $\lambda_{0},\lambda_{1},\cdots ,\lambda_{10}$ is the model coefficient, $\varepsilon ∼N(0,\sigma^{2})$ is the random disturbance.

#### 3.2.4 Mediating effect model of lifestyle choices on health

When the variables of lifestyle (i.e., mediating variables) are incorporated into the independent variables of Model 4, the mediating effect model is derived as follows:


$$\begin{array}{l} \text{Health}=\beta_{0j}+\gamma_{j}\text{Lifestyl}e_{j}+\beta_{1j}\text{AirP}+\beta_{2j}\text{WaterP}+\\ \beta_{3j}\text{ExtWea}+\beta_{4j}\text{VehExh}+\beta_{5j}\text{PesFer}+\\ \beta_{6j}\text{GenCr}+\beta_{7j}\text{FoSaf}+\beta_{8j}\text{LivEnv}+\\ \beta_{9j}\text{VegFru}+\beta_{10j}\text{PubFac}+\varepsilon_{j}^{\prime } \end{array}$$ (5)

where $\text{Lifestyl}e_{1},\;\text{Lifestyl}e_{2},\;\text{Lifestyl}e_{3},\;\text{Lifestyl}e_{4},\;\text{Lifestyl}e_{5},\;\text{Lifestyl}e_{6},\;\text{Lifestyl}e_{7}$ , and $\text{Lifestyl}e_{7}\;\;$ are lifestyle choice variables, which are also the mediating variables of the model; the interpretations of other variables are shown in Table 1.

## 4 Results and analysis

### 4.1 Descriptive statistics analysis

Based on the availability of data,^5^ this study selected 10 environmental perception variables (AirP, WaterP, ExtWea, VehExh, PesFer, GenCr, FoSaf, LivEnv, VegFru, PubFac) and seven lifestyle variables (Lifestyle_1_–Lifestyle_7_) with 2006 samples. The specific interpretations are shown in Table 1. The results of the reliability test show that Cronbach’s coefficient of the environmental perception variables is 0.778, and Cronbach’s coefficient of the lifestyle variables is 0.721, indicating that the selected scales have a high level of internal consistency reliability.

The results of the descriptive statistics show (as shown in Table 1): In the dimension of environmental perception, the mode of residents’ perception levels of air pollution, water pollution and extreme weather is all 1, and the mean value is approximately 2, indicating that most residents hold an optimistic attitude towards the environmental safety of their place of residence. The mode of the perception of the hazards of vehicle exhaust is 3, showing that the public has certain concerns about this type of pollution; the situations of the perception of the hazards of the use of pesticides and fertilizers and genetically modified crops are similar, indicating that residents are relatively sensitive to environmental risks related to agricultural production. In addition, both the mode and the mean value of the perception of living resources such as places for physical exercise, the supply of fruits and vegetables, and the public facilities that can be enjoyed are close to 4, indicating that residents pay high attention to basic living security.

In the dimension of lifestyle, the survey data on physical activities, physical exercises and work regularity show that residents generally have less physical exercise, and there are irregularities in their work and rest schedules. The mode of the perception of social fairness is 4, with a mean value of approximately 3.38, indicating that most residents have a positive evaluation of social fairness. The statistical results of sleep duration, relaxation and rest, and social and entertainment activities also concentrate around 4, indicating that residents’ states in these aspects of life tend to be normal.

Among the 10 environmental perception variables, the sample data exhibit the largest standard deviation in residents’ perception of food safety, with a value of 1.7932. This result indicates that significant disparities exist in people’s perception of food safety across different regions. Regarding the mediating variables, the data distribution of the lifestyle dimension—“residents engaging in physical activities that induce sweating for a minimum of 20 min”—exhibits relatively high dispersion, as evidenced by the largest standard deviation of 1.5909. Regarding the data of the explained variable “physical health status,” its mode is 5, mean is 4.0583, and standard deviation is 0.6378. This result indicates that the vast majority of respondents have an excellent physical health status.

### 4.2 The influence results of environmental perception on lifestyles based on Model 1

Based on the maximum likelihood estimation method, we carried out parameter estimation for Model 1, and the results are shown in Table 2. Taking the dependent variable Lifestyle_1_ (representing “the duration of physical activities with a faster breathing rate than the general situation per week”) in the mediating variable model as the research object, it was set as the reference group of the multinomial Logit model (assigned a value of 0). The study found that: for example, when *j* = 1, the positive influencing factors include air pollution perception (AirP), extreme weather perception (ExtWea), awareness of the harm of genetically modified crops (GenCr), the environment for physical exercise (LivEnv), and the supply of vegetables and fruits (VegFru), which means that the improvement of these environmental perception variables will encourage individuals to increase the time of physical activities; while variables such as water pollution perception (WaterP), vehicle exhaust perception (VehExh), awareness of the harm of pesticides and fertilizers (PesFer), food safety concerns (FoSaf), and the sense of identity with public facilities (PubFac) have a negative correlation with Lifestyle_1_. That is, when the perception levels of the above factors increase, the duration of high-intensity physical activities participated by individuals may be shortened.

**Table 2:** Regression results based on Model 1.

Variable	Sample size	*j* = 1 (reference group)		*j* = 2	*j* = 3	*j* = 4	*j* = 5	*j* = 6	*j* = 7
Const		−0.9736^***^ (0.600)	0	−0.069^*^ (0.534)	−3.7155^**^ (1.213)	0.3464^**^ (1.209)	0.1103^**^ (3.112)	0.1841^**^ (1.539)	−1.5508^*^ (0.792)
AirP	2006	0.0148^***^ (0.088)	0	0.049^*^ (0.079)	−0.0341^**^ (0.184)	−0.0870^**^ (0.168)	−0.3305^**^ (0.501)	0.0464^**^ (0.233)	−0.1305^*^ (0.121)
WaterP	2006	−0.1108^***^ (0.087)	0	−0.037^*^ (0.078)	−0.1787^**^ (0.191)	−0.2036^**^ (0.176)	0.0987^**^ (0.460)	−0.0234^**^ (0.236)	−0.0496^*^ (0.121)
ExtWea	2006	0.0699^***^ (0.068)	0	0.011^*^ (0.063)	0.0001^**^ (0.139)	0.1412^**^ (0.142)	−0.3899^**^ (0.358)	0.1149^**^ (0.186)	0.0139^*^ (0.094)
VehExh	2006	−0.1130^***^ (0.089)	0	−0.043^*^ (0.080)	−0.1831^**^ (0.175)	0.1874^**^ (0.181)	−0.0860^**^ (0.541)	0.3352^**^ (0.232)	−0.0170^*^ (0.119)
PesFer	2006	−0.0885^***^ (0.084)	0	0.011^*^ (0.076)	0.2206^**^ (0.171)	0.0945^**^ (0.173)	0.6035^**^ (0.524)	0.0270^**^ (0.220)	0.0544^*^ (0.113)
GenCr	2006	0.0850^***^ (0.085)	0	0.040^*^ (0.076)	−0.0037^**^ (0.168)	−0.2861^**^ (0.173)	0.8530^**^ (0.531)	0.0545^**^ (0.224)	0.1611^*^ (0.114)
FoSaf	2006	−0.0530^***^ (0.040)	0	−0.027^*^ (0.036)	−0.0174^**^ (0.079)	0.0316^**^ (0.083)	−0.4102^**^ (0.276)	0.0426^**^ (0.107)	0.1516^*^ (0.053)
LivEnv	2006	0.1350^***^ (0.074)	0	0.036^*^ (0.067)	0.3593^**^ (0.174)	−0.0011^**^ (0.151)	−0.2049^**^ (0.406)	−0.0542^**^ (0.185)	−0.1035^*^ (0.093)
VegFru	2006	0.1743^***^ (0.092)	0	−0.083^*^ (0.082)	−0.1157^**^ (0.181)	0.0540^**^ (0.185)	0.7289^**^ (0.417)	0.0052^**^ (0.238)	−0.0459^*^ (0.120)
PubFac	2006	−0.2344^***^ (0.057)	0	0.031^*^ (0.052)	−0.0151^**^ (0.115)	−0.0650^**^ (0.122)	0.3385^**^ (0.409)	0.0387^**^ (0.154)	−0.0113^*^ (0.078)

*, **, and *** indicate statistical significance at the levels of *p* < 0.10, *p* < 0.05, and *p* < 0.01, respectively. The values in parentheses are robust standard errors.

Further analysis reveals that the influence effects of various environmental perception variables on different mediating variables show significant differences. When *j* = 2, the dependent variable Lifestyle_2_ is set as “whether you often engage in physical exercise that makes you sweat or breathe faster for at least 20 min,” at the 10% significance level, the model parameters of six variables, namely air pollution perception, extreme weather perception, awareness of the harm of pesticides and fertilizers, awareness of the harm of genetically modified crops, the environment for physical exercise, and the sense of identity with public facilities, are significantly positive. This indicates that these factors can positively promote regular physical exercise behavior. While the parameters of four variables, namely water pollution perception, vehicle exhaust perception, food safety concerns, and the supply of vegetables and fruits, are significantly negative, suggesting that they have an inhibitory effect on the willingness to engage in physical exercise.

When *j* = 3, the “the regularity of work schedule arrangements in daily life” is taken as the dependent variable Lifestyle_3_, at the 5% significance level, 70% of the 10 environmental perception variables show a negative impact. Only the parameters of three variables, namely extreme weather perception, awareness of the harm of pesticides and fertilizers, and the environment for physical exercise, are significantly positive, meaning that most environmental factors may interfere with the regularity of work schedules.

When *j* = 6, the dependent variable Lifestyle_6_ is “whether you often take a rest and relax in your free time,” under the constraint of the 5% significance level, only the model parameters of water pollution perception and the perception of the suitability for physical exercise within 1 km of the place of residence are significantly negative, and the remaining variables all show a positive promoting effect.

For the detailed parameter estimation results under the settings of other lifestyle variables, please refer to Table 2. The above analysis reveals the heterogeneous characteristics of the influence of environmental perception factors on different dimensions of lifestyle. Based on the above results, Hypothesis H1 is validated.

### 4.3 Analysis based on the multinomial Logit Models 2, 3

#### 4.3.1 Comprehensive analysis of lifestyle (mediating variable)

It should be pointed out that in the process of exploring the influence mechanism of environmental perception on physical health status, the mediating variable—lifestyle—plays an important role. Therefore, we use multinomial Logit Models 2, 3 to quantify this selection probability, in addition, some figures, tables, and random sampling cases are jointly employed to demonstrate the validity of the quantized results. During the model construction process, the first group is selected as the reference group, and its model parameters are set to 0. Based on the regression results in Table 2, the coefficients of the utility functions of Models 2, 3 are determined. By calculating the choice probabilities of 2006 samples in the scenarios of the mode and the mean value, the universality of the research conclusions is enhanced.

**Figure 2:**
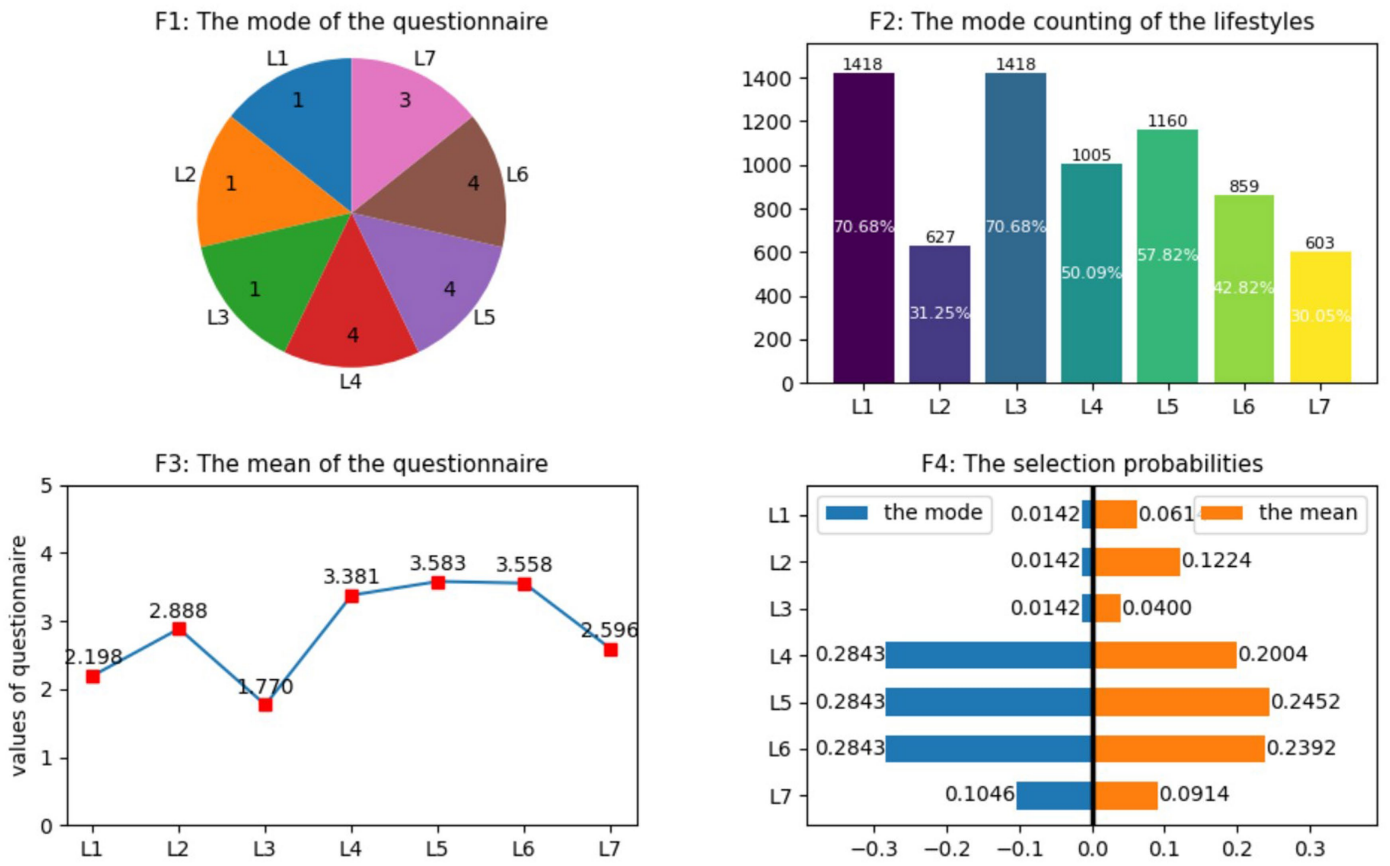
Statistical analysis of residents’ lifestyle and choice intention of multiple Logit models. And L1 represents the length of time on physical activities that make the breath faster than usual each week; L2 denotes the frequency of engaging in physical exercise that makes you sweat or breathe faster for at least 20 min; L3 stands for the regularity of work schedule on weekdays; L4 expresses the perception level of social fairness; L5 means the actual sleep duration on typical workdays; L6 indicates whether an individual engages in rest and relaxation during leisure time, and L7 denotes the frequency of social and recreational activities with friends. The detailed information is shown in Table 1.

As shown in F1 of Figure 2, the mode statistical results of the seven lifestyles reveal the typical characteristics of residents’ living behaviors. The mode of Lifestyle_1_ is 1, corresponding to a sample proportion of 70.68% (1,418 people), indicating that more than 70% of the respondents have less than 30 min of high-intensity physical activities (with a significantly increased breathing rate) per week, which reflects from the side that under the background of the popularization of mechanized labor in China, the participation rate of residents in physical activities is relatively low. Based on the calculation of the multinomial Logit model, the choice probabilities of this lifestyle in the mode and mean value scenarios are 0.0142 and 0.0614 respectively, showing that residents have a weak willingness to maintain their health through physical labor.

The mode of Lifestyle_2_ is also 1, corresponding to 31.25% of the samples, which means that approximately one-third of the respondents rarely engage in sweating or high-intensity exercises lasting more than 20 min. Its choice probabilities under the mode and the mean value are 0.0142 and 0.1224 respectively, indicating that the awareness of residents to actively participate in physical exercise urgently needs to be improved. The mode of Lifestyle_3_ is 1 (accounting for 70.68%), reflecting that most of the respondents lack regularity in their work; the choice probabilities calculated by the model are 0.0142 (mode) and 0.04 (mean value) respectively, suggesting that residents generally tend to avoid an irregular work pattern.

In contrast, the modes (4, 4, 4, 3) and means (3.381, 3.583, 3.558, 2.596) of Lifestyle_4_–Lifestyle_7_ are relatively high, indicating that residents have a good perception of social fairness and show positive performance in aspects such as sleep duration, leisure and relaxation, and social entertainment. The corresponding results of the multinomial Logit model show that the choice probabilities of these lifestyles are significantly higher than those of the previous three, confirming that residents are more inclined to relieve work pressure through sufficient sleep, leisure and relaxation, and social activities (Figure 2: F1–F4).

Specifically, in the 4th subfigure F4 of Figure 2, the multinomial Logit model was employed to compute the selection probabilities of the seven lifestyles based on the mode and mean value of the variables. As illustrated by the results, under the mean-value scenario, the two lifestyles with the highest selection probabilities are “sleep duration on workdays” with 0.2452 and “rest and leisure time” with 0.2392. Therefore, Hypothesis H2 is supported.

#### 4.3.2 Case testing and analysis based on random sampling

In Part (A) of Section 4.3, we analyzed the selection probability of residents’ lifestyles based on the full sample. The purpose of Part (B) in this section is to test and verify the results of Part (A). The sampling sources are regions with relatively high and relatively low economic development level, such as Beijing and Ningxia, Jiangsu and Guangxi, Zhejiang and Henan. Although the sample size of the sampling is small (3 individuals sampled in each group), the comparison is conducted in a random manner with three groups of data selected. Therefore, according to the principle of statistical inference, the test results have high credibility.

Based on the analysis of the overall sample, this part focuses on regional differences. Three individual questionnaires are randomly selected from the 10 environmental perception variables of the two sample sources, Beijing and Ningxia (the details of the samples are shown in Table 3). By comparing the differences in lifestyle choices of individuals in different regions under the same environmental perception variables, the impact of the level of economic development on residents’ lifestyle preferences is explored.

**Table 3:** Individual questionnaire cases in Beijing and Ningxia.

Variable	AirP	WaterP	ExtWea	VehExh	PesFer	GenCr	FoSaf	LivEnv	VegFru	PubFac
Source region	Beijing	Beijing	Beijing	Beijing	Beijing	Beijing	Beijing	Beijing	Beijing	Beijing
Ind. 1	2	4	1	5	5	5	3	4	4	4
Ind. 2	5	3	3	4	3	5	2	2	5	1
Ind. 3	1	2	1	3	3	4	2	4	4	3

Variable	AirP	WaterP	ExtWea	VehExh	PesFer	GenCr	FoSaf	LivEnv	VegFru	PubFac
Source region	Ningxia	Ningxia	Ningxia	Ningxia	Ningxia	Ningxia	Ningxia	Ningxia	Ningxia	Ningxia
Ind. 1	3	5	2	3	3	4	2	2	4	4
Ind. 2	2	1	2	3	2	3	5	2	5	5
Ind. 3	1	1	1	4	3	2	5	5	5	5

**Figure 3:**
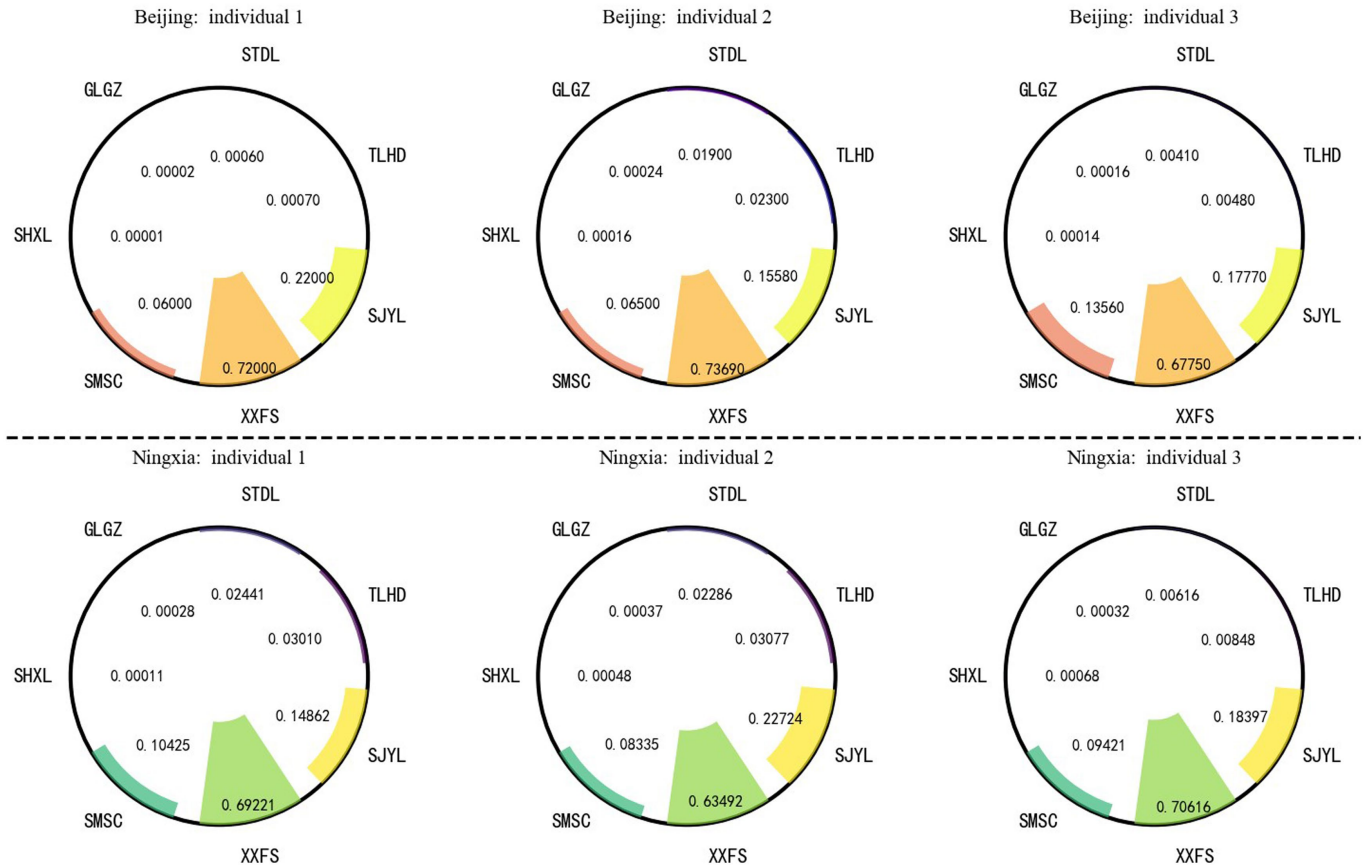
Probability distribution of the choices of lifestyle by residents in Beijing and Ningxia. TLHD represents the probability of choosing physical activities; STDL represents the probability of choosing physical exercises; GLGZ represents the probability of choosing regular work; SHXL represents the probability of choosing to pay attention to the psychological state of social fairness or unfairness; SMSC represents the probability of choosing the duration of sleep in terms of lifestyle; XXFS represents the probability of choosing to rest and relax during free time; SJYL represents the probability of choosing the frequency of social entertainment during free time.

Based on the calculation Equations 2, 3 of the multinomial Logit model, the probabilities of lifestyle choices for a total of 6 samples from Beijing and Ningxia were calculated in Figure 3. The study found that the three individuals in Beijing showed a high degree of consistency in the probability distribution of lifestyle choices: the probability of “often taking a rest and relaxing in their free time” was the highest, reaching 0.72; the probability of “engaging in social and entertainment activities with friends (such as visiting each other, having meals, playing cards, etc.)” was the second highest, indicating that residents in Beijing are more inclined to enrich their leisure time through leisure and relaxation and social activities; while the probability of “psychological perception of social fairness” was the lowest, only 0.0001, reflecting that this group has relatively limited attention to social fairness issues.

The three individuals of Ningxia in Figure 3 showed probability distribution characteristics similar to those of the samples in Beijing: the probabilities of choosing rest, relaxation, social and entertainment activities were high, while the attention to social fairness was low. The comparison between different regions shows that although Beijing is a developed region and Ningxia is located in the western part of China, there is no significant difference in the preferences for lifestyle choices between the residents of the two places. This result indicates that residents in regions with different levels of economic development in China have convergence in their lifestyle choices, and their action orientations may be less directly restricted by regional economic conditions.

**Figure 4:**
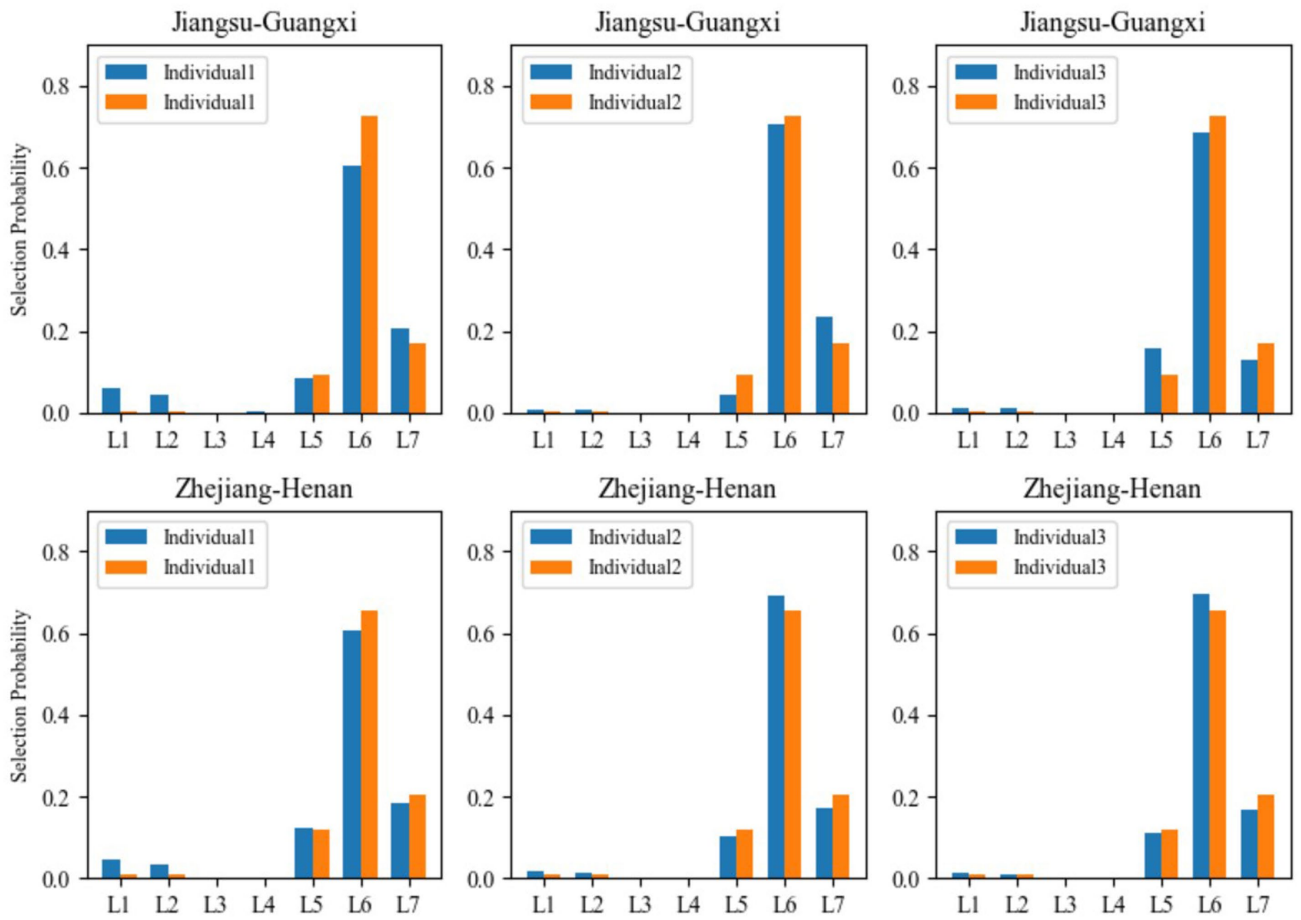
Comparison chart of trends in lifestyle choices. L1 represents the length of time on physical activities that make the breath faster than usual each week; L2 denotes the frequency of engaging in physical exercise that makes you sweat or breathe faster for at least 20 min; L3 stands for the regularity of work schedule on weekdays; L4 expresses the perception level of social fairness; L5 means the actual sleep duration on typical workdays; L6 indicates whether an individual engages in rest and relaxation during leisure time, and L7 denotes the frequency of social and recreational activities with friends. The detailed information is shown in Table 1.

To enhance the robustness of the research findings, we employed more sample cases to substantiate the universality of the results. From the perspective of economic development, based on data from Jiangsu and Guangxi, Zhejiang and Henan, we randomly selected three samples from each of these four provinces (totaling 12 samples) and applied the multinomial Logit model to calculate their selection probabilities. This approach was used to analyze whether residents from different geographical locations exhibit heterogeneity in their lifestyle choices. To facilitate easier comparison and intuitive presentation of the results, we visualized the findings in figures. In Figure 4, the lifestyle choices of the six sample individuals from Jiangsu and Guangxi, as well as the six from Zhejiang and Henan, are predominantly concentrated in “L5, L6, L7,” which represent leisure and relaxation, sufficient sleep, and social activities. Therefore, the results presented in Figures 3, 4 further corroborate the robustness and universality of this study’s findings.

That is to say, among the given seven lifestyle options, “leisure and relaxation,” “sufficient sleep,” and “social interaction activities” are the most frequent choices made by residents to maintain their physical health. Consequently, the results confirm Hypothesis H2.

### 4.4 Influence effects of physical health

#### 4.4.1 The influence effects based on Model 4

In the second column of Table 4, the model coefficients show that different environmental perception variables have differential impacts on residents’ physical health. The positive influencing variables include WaterP (perception of water pollution), ExtWea (perception of extreme weather), PesFer (awareness of the harm of pesticides and fertilizers), GenCr (awareness of the harm of genetically modified crops), FoSaf (perception of food safety), and VegFru (perception of the availability of fresh vegetables and fruits). That is, residents effectively mitigate health risks by proactive measures against water pollution and extreme weather, cautious selection of foods to address pesticide residues and genetically modified ingredients, and enhance well-being by utilizing locally available fruits and vegetables.

**Table 4:** Regression results based on Models 4, 5.

Dependent variable: physical health status								
Variable	Model 4	Model 5 *j* = 1	Model 5 *j* = 2	Model 5 *j* = 3	Model 5 *j* = 4	Model 5 *j* = 5	Model 5 *j* = 6	Model 5 *j* = 7
Const	3.9153 (0.117)	3.9193 (0.119)	3.9124 (0.121)	3.9252 (0.119)	3.9283 (0.129)	4.1051 (0.125)	3.9838 (0.130)	3.9372 (0.123)
AirP	−0.0182^***^ (0.020)	−0.0182^**^ (0.020)	−0.0183^**^ (0.020)	−0.0184^**^ (0.020)	−0.0183^**^ (0.020)	−0.0180^**^ (0.020)	−0.0179^**^ (0.020)	−0.0187^**^ (0.020)
WaterP	0.0004^***^ (0.019)	0.0002^**^ (0.019)	0.0004^**^ (0.0220)	0.0006^**^ (0.019)	0.0001^**^ (0.019)	−0.0009^**^ (0.019)	0.0004^**^ (0.019)	0.0002^**^ (0.019)
ExtWea	0.0220^***^ (0.015)	0.0220^**^ (0.015)	0.0220^**^ (0.015)	0.0217^**^ (0.015)	0.0221^**^ (0.015)	0.0223^**^ (0.015)	0.0222^**^ (0.015)	0.0221^**^ (0.015)
VehExh	−0.0062^***^ (0.019)	−0.0063^**^ (0.019)	−0.0061^**^ (0.019)	−0.0064^**^ (0.019)	−0.0061^**^ (0.019)	−0.0038^**^ (0.019)	−0.0049^**^ (0.019)	−0.0060^**^ (0.019)
PesFer	0.0087^***^ (0.018)	0.0087^**^ (0.018)	0.0087^**^ (0.018)	0.0088^**^ (0.018)	0.0088^**^ (0.018)	0.0083^**^ (0.018)	0.0086^**^ (0.018)	0.0085^**^ (0.018)
GenCr	0.0099^***^ (0.018)	0.0101^**^ (0.018)	0.0099^**^ (0.018)	0.0100^**^ (0.018)	0.0098^**^ (0.018)	0.0095^**^ (0.018)	0.0096^**^ (0.018)	0.0100^**^ (0.018)
FoSaf	0.0083^***^ (0.010)	0.0084^**^ (0.010)	0.0083^**^ (0.010)	0.0081^**^ (0.010)	0.0084^**^ (0.010)	0.0065^** **^ (0.010)	0.0085^**^ (0.010)	0.0084^**^ (0.010)
LivEnv	−0.0027^***^ (0.016)	−0.0026^**^ (0.016)	−0.0028^**^ (0.016)	−0.0025^**^ (0.016)	−0.0028^**^ (0.016)	0.0006^**^ (0.016)	−0.0037^**^ (0.016)	−0.0029^**^ (0.016)
VegFru	0.0296^***^ (0.020)	0.0298^**^ (0.020)	0.0296^**^ (0.020)	0.0296^**^ (0.020)	0.0295^**^ (0.020)	0.0275^**^ (0.020)	0.0297^**^ (0.020)	0.0292^**^ (0.020)
PubFac	−0.0124^***^ (0.012)	−0.0126^**^ (0.013)	−0.0124^**^ (0.012)	−0.0124^**^ (0.012)	−0.0124^**^ (0.012)	−0.0109^**^ (0.012)	−0.0124^**^ (0.012)	−0.0125^**^ (0.012)
Lifestyle_1_		−0.0253^**^ (0.010)						
Lifestyle_2_			−0.0236^**^ (0.009)					
Lifestyle_3_				−0.0455^**^ (0.011)				
Lifestyle_4_					0.0237^**^ (0.015)			
Lifestyle_5_						0.0353^**^ (0.014)		
Lifestyle_6_							0.0198^**^ (0.015)	
Lifestyle_7_								0.0074^**^ (0.011)

*, **, and *** indicate statistical significance at the levels of *p* < 0.10, *p* < 0.05, and *p* < 0.01, respectively. The values in parentheses are robust standard errors.

Relatively speaking, the negative influencing variables include AirP (perception of air pollution), VehExh (perception of vehicle exhaust pollution), LivEnv (perception of the suitability of physical exercise places within 1 km), and PubFac (perception of public facilities at the place of residence), indicating that the enhancement of these factors will not directly promote the improvement of residents’ health conditions. Regarding the negative regression coefficients, it is logically consistent: when individuals perceive more severe air pollution, vehicle emissions, or consider exercise facilities to be inconvenient or poorly maintained, such negative environmental exposures and perceptions (e.g., by reducing outdoor activities) can harm physical health. Therefore, it is natural for these perceptual variables to exhibit a negative correlation with physical health levels.

Thus, the results from the above presentation provide evidence for Hypothesis H3.

#### 4.4.2 The influence effects based on Model 5

In the regression results of Model 5 (the 3rd column to the ninth column in Table 4, namely *j* = 1 to *j* = 7), environmental perception variables show different health influence effects. The coefficients of ExtWea (perception of extreme weather), PesFer (awareness of the harm of pesticides and fertilizers), GenCr (awareness of the harm of genetically modified crops), FoSaf (perception of food safety), and VegFru (perception of the supply of vegetables and fruits) are positive, indicating that the improvement of these dimensions of environmental perception has a positive promoting effect on health. While the index coefficients of AirP (perception of air pollution), VehExh (perception of vehicle exhaust pollution), and PubFac (perception of public facilities) are all negative, suggesting that residents’ negative perceptions of air pollution and public facilities will suppress their health levels. Alternatively, when residents perceive the presence of air pollution, vehicle exhaust pollution, and poor exercise facilities, these factors are detrimental to physical health. Therefore, it is entirely reasonable that the model parameters for these perceptual variables exhibit negative values.

When the mediating variables are successively added as independent variables into Model 5, the model coefficients of Lifestyle_1_ (duration of high-intensity physical activities), Lifestyle_2_ (regular physical exercise), and Lifestyle_3_ (work regularity) are negative, revealing the potential health hazards of modern lifestyles. The results show that contemporary people have insufficient participation in physical activities, a low frequency of physical exercise, and more than half of the individuals have irregular working hours. These factors jointly lead to a negative impact on their health. On the contrary, the coefficients of Lifestyle_4_ (perception of social fairness), Lifestyle_5_ (sleep duration on working days), Lifestyle_6_ (relaxation in free time), and Lifestyle_7_ (social and entertainment activities) are positive, which reflect the health promotion mechanisms from the psychological and behavioral aspects respectively: the perception of fairness strengthens individuals’ social trust and sense of security, thus improving life satisfaction; sufficient sleep effectively restores the body’s functions and improves cognitive and emotional stability; leisure and relaxation can relieve work pressure and maintain the balance of the body and mind; social and entertainment activities enhance individuals’ health resilience through emotional support and stress release. Based on the above results and analysis, Hypothesis H4 is naturally corroborated.

### 4.5 Test and analysis of the mediation effect and transmission mechanism

#### 4.5.1 Direct test and analysis of mediating effect

**Figure 5:**
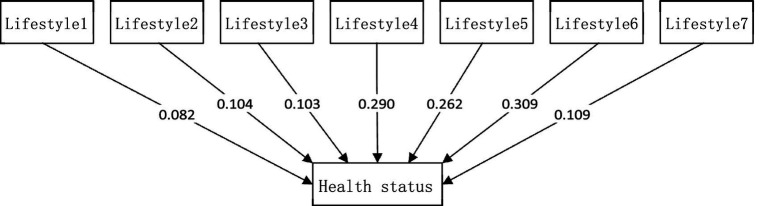
Influence effects of lifestyle on physical health status.

In Model 5, by stripping away the multi-dimensional environmental perception variables, the analysis focuses on the influence effects of lifestyle on physical health status. Based on the questionnaire data of the seven lifestyles (Lifestyle_1_ to Lifestyle_7_) and health status, the OLS regression method is used to complete the estimation of the model parameters. In order to more intuitively present the influence degree of each variable, the research results are presented in a visual form in Figure 5.

As shown in Figure 5, among various lifestyle factors, the “monthly frequency of rest and relaxation” (Lifestyle_6_) has the most significant impact on health. This means that regular rest and relaxation is an important way to relieve mental fatigue and adjust physical and mental states. In contrast, the impact of “weekly duration of physical activity” (Lifestyle_1_) is relatively weak, which indicates that most residents tend to choose low-intensity daily physical activities.

#### 4.5.2 Analysis of the transmission mechanism of mediating effect

In this part, the Baron & Kenny step-by-step method was adopted to test the action mechanism of the mediating variable (residents’ lifestyle). The study found (as shown in Table 5) that the mediating effect of lifestyle shows a significant polarization characteristic.

**Table 5:** Results of the Baron & Kenny stepwise method test for the mediating effect.

Effect type	Lifestyle_1_	Lifestyle_2_	Lifestyle_3_	Lifestyle_4_	Lifestyle_5_	Lifestyle_6_	Lifestyle_7_
Total effect	−0.0368	−0.0368	−0.0368	0.0427	0.0395	0.0248	0.0209
Direct effect	−0.0367	−0.0369	−0.0371	0.0370	0.0322	0.0223	0.0211
Indirect effect	−0.0001	0.0001	0.0003	0.0057	0.0073	0.0025	−0.0002
Significance	Not significant	Not significant	Not significant	Significant	Significant	Significant	Relatively significant
Controlled independent variable	Controlled	Controlled	Controlled	Controlled	Controlled	Controlled	Controlled

In the positive mediating path, psychological and leisure-related variables, mainly including the perception of social fairness, sleep duration, the frequency of leisure and relaxation, and the participation degree in social activities, exhibit a positive promoting effect. The research data shows that the effect values of the above variables are all positive, indicating that good environmental perception can significantly enhance residents’ cognitive awareness of social fairness, ensure sleep quality, and promote the development of leisure and social activities, ultimately having a positive impact on physical health. The mediating effect of perceived social equity and sleep duration is particularly prominent, which reflects that environmental improvement can significantly enhance residents’ health benefits.

In terms of the negative mediating path, variables related to physical activities and life regularity, mainly including the duration of high-intensity physical activities, the frequency of sweating-type exercise, and work regularity, show a significant negative mediating effect. The research results show that both the total effect and the direct effect between the level of environmental perception and the above variables are negative values. This indicates that when individuals perceive a deterioration in environmental quality, the likelihood of their participating in regular physical exercise and maintaining an orderly work rhythm is significantly reduced, which in turn has a negative impact on physical health, forming a transmission path of “environmental pressure—behavioral disorder—health deterioration.”

### 4.6 Robustness testing and analysis of core conclusions

To test whether the main research conclusions of this paper are robust, we select the gender (denoted by Gend ), educational level (indicated by EduLev ), and economic income (expressed by EcoInc ) as the core explanatory variables and add them to Models 1, 4, 5 respectively. This selection of three variables is based on the social-ecological model theory and data availability. The new models constructed are shown in Models 6, 7, 8.


$$\begin{array}{c} \text{Lifestyl}e_{j}=\mu_{0j}+\mu_{1j}\text{AirP}+\mu_{2j}\text{WaterP}+\mu_{3j}\text{ExtWea}\\ +\mu_{4j}\text{VehExh}+\mu_{5j}\text{PesFer}+\mu_{6j}\text{GenCr}+\\ \mu_{7j}\text{FoSaf}+\mu_{8j}\text{LivEnv}+\mu_{9j}\text{VegFru}+\\ \mu_{10j}\text{PubFac}+\mu_{11j}\text{Gend}+\\ \mu_{12j}\text{EduLev}+\mu_{13j}\text{EcoInc}+\varepsilon_{j} \end{array}$$ (6)

$$\begin{array}{c} \text{Health}=\gamma_{0}+\gamma_{1}\text{AirP}+\gamma_{2}\text{WaterP}+\gamma_{3}\text{ExtWea}\\ +\gamma_{4}\text{VehExh}+\gamma_{5}\text{PesFer}+\gamma_{6}\text{GenCr}+\gamma_{7}\text{FoSaf}\\ +\gamma_{8}\text{LivEnv}+\gamma_{9}\text{VegFru}+\gamma_{10}\text{PubFac}+\gamma_{11}\text{Gend}\\ +\gamma_{12}\text{EduLev}+\gamma_{13}\text{EcoInc}+\varepsilon \end{array}$$ (7)

$$\begin{array}{c} \text{Health}=\rho_{0j}+\rho_{j}\;\text{Lifestyl}e_{j}+\rho_{1j}\text{AirP}+\rho_{2j}\text{WaterP}\\ +\rho_{3j}\text{ExtWea}+\rho_{4j}\text{VehExh}+\\ \rho_{5j}\text{PesFer}+\rho_{6j}\text{GenCr}+\rho_{7j}\text{FoSaf}+\\ \rho_{8j}\text{LivEnv}+\rho_{9j}\text{VegFru}+\rho_{10j}\text{PubFac}\\ +\rho_{11j}\text{Gend}+\rho_{12j}\text{EduLev}+\rho_{13j}\text{EcoInc}+\varepsilon_{j}^{'} \end{array}$$ (8)

where j=1,2,⋯,7 , Gend denotes gender, EduLev represents educational level, EcoInc indicates economic income and the meanings of other symbols are explained in (1), (4) and (5).

Table 6 presents the regression results of Model 6. Comparing the signs (positive/negative) of the regression coefficients with those of the corresponding variables in Model 1, we observe that when *j* = 1, only the coefficient sign of FoSaf is inconsistent, resulting in a 90% consistency rate for coefficient signs. When *j* = 2, the consistency rate reaches 90%. At *j* = 3, the rate drops to 60%, but it returns to 90% for *j* = 4. For *j* = 5, the consistency rate is only 20%, while for *j* = 6 and *j* = 7, it reaches 80%, respectively. Overall, the consistency rate of regression coefficient signs is 72.86% (excluding the constant term). These findings indicate that Model 1 exhibits reasonably good robustness.

**Table 6:** Regression results based on Model 6.

Variable	*j* = 1 (reference group)	*j* = 2	*j* = 3	*j* = 4	*j* = 5	*j* = 6	*j* = 7
Const	1.9804^***^ (0.327)	3.2862^*^ (0.359)	1.8182^**^ (0.283)	3.6063^**^ (0.218)	3.1324^**^ (0.225)	3.3709^**^ (0.211)	3.0712^*^ (0.284)
AirP	0.0067^***^ (0.043)	0.0548^*^ (0.047)	−0.0306^**^ (0.037)	−0.0157^**^ (0.028)	0.0038^**^ (0.029)	0.0168^**^ (0.028)	−0.0631^*^ (0.037)
WaterP	−0.0590^***^ (0.042)	−0.0361^*^ (0.046)	0.0437^**^ (0.036)	−0.0600^**^ (0.028)	−0.0264^**^ (0.029)	−0.0010^**^ (0.027)	−0.0165^*^ (0.037)
ExtWea	0.0148^***^ (0.034)	0.0144^*^ (0.037)	−0.0477^**^ (0.029)	0.0306^**^ (0.022)	0.0066^**^ (0.023)	0.0085^**^ (0.022)	0.0192^*^ (0.029)
VehExh	−0.0923^***^ (0.043)	−0.0149^*^ (0.048)	−0.0417^**^ (0.038)	0.0119^**^ (0.029)	0.0450^**^ (0.030)	0.0675^**^ (0.028)	0.0291^*^ (0.038)
PesFer	−0.0118^***^ (0.041)	−0.0083^*^ (0.045)	0.0167^**^ (0.036)	0.0486^**^ (0.027)	−0.0083^**^ (0.028)	−0.0008^**^ (0.027)	−0.0195^*^ (0.036)
GenCr	0.0759^***^ (0.041)	0.0099^*^ (0.045)	0.0221^**^ (0.036)	−0.0343^**^ (0.027)	−0.0107^**^ (0.028)	−0.0139^**^ (0.027)	0.0108^*^ (0.036)
FoSaf	0.0522^***^ (0.023)	−0.0486^*^ (0.025)	−0.0320^**^ (0.020)	0.0286^**^ (0.015)	−0.0319^**^ (0.016)	0.0105^**^ (0.015)	0.0204^*^ (0.020)
LivEnv	0.0774^***^ (0.036)	0.0087^*^ (0.039)	0.0368^**^ (0.031)	−0.0148^**^ (0.024)	0.0604^**^ (0.025)	−0.0467^**^ (0.023)	−0.0158^*^ (0.031)
VegFru	0.0809^***^ (0.044)	−0.0380^*^ (0.048)	0.0016^**^ (0.038)	−0.0177^**^ (0.029)	−0.0396^**^ (0.030)	0.0066^**^ (0.028)	−0.0556^*^ (0.038)
PubFac	−0.1250^***^ (0.028)	0.0291^*^ (0.031)	−0.0007^**^ (0.025)	−0.0157^**^ (0.019)	0.0257^**^ (0.020)	−0.0025^**^ (0.018)	−0.0117^*^ (0.025)
Gend	0.0139^***^ (0.066)	−0.0018^*^ (0.072)	−0.0420^**^ (0.057)	−0.0202^**^ (0.044)	0.0896^**^ (0.045)	0.0039^**^ (0.043)	−0.0262^*^ (0.057)
EduLev	0.0497^***^ (0.035)	−0.0298^*^ (0.039)	0.0145^**^ (0.031)	−0.0398^**^ (0.024)	0.0123^**^ (0.024)	−0.0120^**^ (0.023)	−0.0449^*^ (0.031)
EcoInc	−0.0271^***^ (0.037)	−0.0450^*^ (0.040)	0.0072^**^ (0.032)	−0.0037^**^ (0.025)	0.0407^**^ (0.025)	0.0260^**^ (0.024)	−0.0027^*^ (0.032)

*, **, and *** indicate statistical significance at the levels of *p* < 0.10, *p* < 0.05, and *p* < 0.01, respectively. The values in parentheses are robust standard errors.

Using 2006 sample data and the maximum likelihood method, the results of Model 7 are derived via the statsmodels library in Python, as presented in Table 7. The regression results of Model 7 in Table 7 show exactly the same sign (positive or negative) as the corresponding coefficients of the second column in Table 4 for the independent variables of Model 4. This means the independent variables have the same effect on the dependent variable in both Models 4, 7. Therefore, when we added the three core independent variables (Gender, Education Level, Economic Income) to Model 4 [that is, Model 4 is transformed into Model 7], their impact on the dependent variable remained consistent. This confirms that Model 4 is robust.

**Table 7:** Regression results based on Model 7.

Variables	Const	AirP	WaterP	ExtWea	VehExh	PesFer	GenCr
Model coefficients	4.0136^**^ (0.144)	−0.0157^**^ (0.019)	0.0003^**^ (0.019)	0.0219^**^ (0.015)	−0.0045^**^ (0.019)	0.0123^**^ (0.018)	0.0106^**^ (0.018)

Variables	FoSaf	LivEnv	VegFru	PubFac	Gend	EduLev	EcoInc
Model coefficients	0.0087^**^ (0.010)	−0.0031^**^ (0.016)	0.0291^**^ (0.019)	−0.0114^**^ (0.012)	−0.0140^**^ (0.029)	−0.0296^**^ (0.016)	−0.0073^**^ (0.016)

*, **, and *** indicate statistical significance at the levels of *p* < 0.10, *p* < 0.05, and *p* < 0.01, respectively. The values in parentheses are robust standard errors.

Table 8 shows the regression results of Model 8. Comparing the signs (positive/negative) of the regression coefficients with those of the corresponding variables in Model 5, we see a complete match when *j* = 1, yielding a 100% consistency rate. For *j* = 2, the sign consistency rate is 90.9%. At *j* = 3, it reaches 100% again, while for *j* = 4, the values in rate is 81.82%. For *j* = 5, *j* = 6, and *j* = 7, the sign consistency remains at 90.9% in each case. Overall, the consistency rate between the corresponding coefficients of the two models is 92.21% (excluding the constant term). These results demonstrate that the regression findings of Model 5 are robust. Parentheses are robust standard errors.

**Table 8:** Regression results based on Model 8.

Variable	*j* = 1	*j* = 2	*j* = 3	*j* = 4	*j* = 5	*j* = 6	*j* = 7
Const	4.0167 (0.145)	4.0116 (0.147)	4.0233 (0.145)	4.0312 (0.153)	4.1856 (0.150)	4.0812 (0.153)	4.0386 (0.148)
AirP	−0.0156^**^ (0.019)	−0.0157^**^ (0.019)	−0.0158^**^ (0.019)	−0.0157^**^ (0.019)	−0.0154^**^ (0.019)	−0.0153^**^ (0.019)	−0.0162^**^ (0.019)
WaterP	0.0002^**^ (0.019)	0.0003^**^ (0.019)	0.0005^**^ (0.019)	−0.0003^**^ (0.019)	−0.0012^**^ (0.018)	0.0002^**^ (0.018)	0.0001^**^ (0.019)
ExtWea	0.0219^**^ (0.015)	0.0219^**^ (0.015)	0.0216^**^ (0.015)	0.0220^**^ (0.015)	0.0223^**^ (0.015)	0.0221^**^ (0.015)	0.0220^*^ (0.015)
VehExh	−0.0047^**^ (0.019)	−0.0045^**^ (0.019)	−0.0048^**^ (0.019)	−0.0045^**^ (0.019)	−0.0021^**^ (0.019)	−0.0032^**^ (0.019)	−0.0060^**^ (0.019)
PesFer	0.0122^**^ (0.018)	0.0123^**^ (0.018)	0.0124^**^ (0.018)	0.0125^**^ (0.018)	0.0118^**^ (0.018)	0.0122^**^ (0.018)	0.0121^**^ (0.018)
GenCr	0.0107^**^ (0.018)	0.0106^**^ (0.018)	0.0107^**^ (0.018)	0.0104^**^ (0.018)	0.0100^**^ (0.018)	0.0103^**^ (0.018)	0.0107^**^ (0.018)
FoSaf	0.0088^**^ (0.010)	0.0087^**^ (0.010)	0.0085^**^ (0.010)	0.0088^**^ (0.010)	0.0069^**^ (0.010)	0.0089^**^ (0.010)	0.0089^**^ (0.010)
LivEnv	−0.0026^**^ (0.016)	−0.0031^**^ (0.016)	−0.0029^**^ (0.016)	−0.0032^**^ (0.016)	0.0002^**^ (0.016)	−0.0041^**^ (0.016)	−0.0032^**^ (0.016)
VegFru	−0.0030^**^ (0.016)	0.0292^**^ (0.019)	0.0291^**^ (0.019)	0.0290^**^ (0.019)	0.0270^**^ (0.019)	0.0293^**^ (0.019)	0.0287^**^ (0.019)
PubFac	−0.0116^**^ (0.013)	−0.0114^**^ (0.012)	−0.0114^**^ (0.012)	−0.0115^**^ (0.012)	−0.0100^**^ (0.012)	−0.0114^**^ (0.012)	−0.0115^**^ (0.012)
Gend	−0.0139^**^ (0.029)	−0.0140^**^ (0.029)	−0.0142^**^ (0.029)	−0.0141^**^ (0.029)	−0.0090^**^ (0.029)	−0.0139^**^ (0.029)	−0.0142 (0.029)
EduLev	−0.0296^**^ (0.016)	−0.0296^**^ (0.016)	−0.0296^**^ (0.016)	−0.0298^**^ (0.016)	−0.0290^**^ (0.016)	−0.0299^**^ (0.016)	−0.0300 (0.016)
EcoInc	−0.0074^**^ (0.016)	−0.0073^**^ (0.016)	−0.0073^**^ (0.016)	−0.0074^**^ (0.016)	−0.0051^**^ (0.016)	−0.0068^**^ (0.016)	−0.0074 (0.016)
Lifestyle_1_	−0.0015^**^ (0.010)						
Lifestyle_2_		0.0006^**^ (0.009)					
Lifestyle_3_			−0.0053^**^ (0.011)				
Lifestyle_4_				−0.0049^**^ (0.015)			
Lifestyle_5_					−0.0549^**^ (0.014)		
Lifestyle_6_						−0.0200^**^ (0.015)	
Lifestyle_7_							−0.0081^**^ (0.011)

*, **, and *** indicate statistical significance at the levels of *p* < 0.10, *p* < 0.05, and *p* < 0.01, respectively.

## 5 Discussion

This study employs quantitative methods to examine the relationships among environmental perception, lifestyle, and residents’ health, using data from the Chinese General Social Survey (CGSS 2021). While the research yields significant academic value, it also presents inherent limitations and areas that necessitate critical reflection. It is worth noting that the selected variables and the acquisition of questionnaire data are both subjective, which impose certain conservatism and limitations on the research results. This should be specifically noted here.

### 5.1 Strengths of the research findings

In terms of academic contribution, the core strengths of this study are manifested in the seamless integration of theoretical frameworks and empirical analysis. Theoretically, it innovatively combines the Social Ecological Model with Protection Motivation Theory (PMT), transcending the simplistic causal relationship of “environmental perception influencing health” to construct a multi-level logical chain: “micro-level individual cognition → meso-level behavioral choices (lifestyle) → macro-level health outcomes.” Specifically, PMT is utilized to elucidate how environmental perception shapes health-related behaviors through two core dimensions: “threat appraisal” (e.g., risk assessments of extreme weather events and food safety hazards) and “coping appraisal” (e.g., enhancing health-related self-efficacy via regular sleep patterns and social engagement). Complementarily, the Social Ecological Model contextualizes these micro-level mechanisms by incorporating the supportive roles of macro-variables, such as community environmental governance and public service provision. This theoretical integration not only reinforces the validity of the research conclusions but also provides an integrated “micro-meso-macro” analytical paradigm for future studies in the field.

Empirically, the triangulation of research methods significantly enhances the robustness of the findings. Firstly, the multinomial Logit model is employed to quantify the probability of lifestyle choices, addressing the inadequacy of traditional linear models in handling “categorical behavioral variables.” This model enables the clear identification of probabilistic associations between distinct dimensions of environmental perception (e.g., positive perceptions of fresh produce availability, concerns about vehicle emissions) and lifestyle indicators (e.g., sleep quality, frequency of social interactions). Similarly, Shivam et al. (26) used the Logit model to study the influence mechanism of daily activities and the surrounding environment on health. Secondly, the mediating effect model clarifies the “intermediary role” of lifestyle, confirming that environmental perception exerts an indirect impact on health through the sequential pathway: “reducing psychological distress → improving behavioral efficacy → adopting healthy lifestyles.” For example, Liu et al. (27) explored physical activity as a mediator of the associations between neighborhood environments and the health status of Chinese older adults during the pandemic. Therefore, our study offers targeted insights for public health interventions, avoiding the myopic focus on either environmental governance or behavioral guidance in isolation.

### 5.2 Discussion on limitations of the research

We primarily discuss the research limitations from the following two aspects. On the one hand, the study exhibits constraints in variable comprehensiveness and measurement precision. Key confounding factors, such as individual-level predisposing conditions (e.g., chronic disease history), were omitted, potentially introducing bias in estimating the causality between environmental perception and lifestyle behaviors. Furthermore, the reliance on self-reported measures for health outcomes inherently incorporates systematic error, while incomplete operationalization of lifestyle constructs (e.g., absence of dietary pattern metrics) limits the robustness of mediating effect analyses. On the other hand, the analytical approach employed a multinomial Logit model on a limited sample (*N* = 2,006), whereas extant methodologies [e.g., ordered Logit models in Zeng and Yang (28)] suggest potential avenues for methodological refinement. Although cross-validation through stratified random sampling of six regions adhered to statistical inference principles, the restricted sample size remains susceptible to estimation inaccuracies arising from low-probability stochastic events.

### 5.3 Extended discussion

Without mediation analysis, a study may lack depth and fail to develop or test behavioral mechanisms theory. Even if regression analysis reveals a significant effect of environmental perception on physical health, the absence of mediation testing (e.g., through lifestyle) leaves the “how” unexplained. Does it operate by promoting/inhibiting physical exercise? Or by altering dietary habits? This critical process mechanism remains a “black box.” Therefore, investigating the relationship between environmental perception and physical health through the mediating variable (lifestyle) is both methodologically justified and substantively important.

### 5.4 Directions for improvement in future research

To address the aforementioned limitations, future studies can be optimized in three key areas. Firstly, data updates and model extensions are recommended: for example, adopting a “two-way fixed effects model” to control for time-invariant individual characteristics. Secondly, variable design should be refined: incorporating individual-level variables such as pre-existing medical conditions and macro-level variables such as healthcare policies, while expanding the measurement dimensions of “lifestyle” to enhance precision. Thirdly, causal identification should be strengthened: employing the “instrumental variable (IV) method”—for instance, using “the number of public parks within a community” as an instrumental variable for environmental perception—to mitigate endogeneity issues between “environmental perception and health,” thereby improving the validity of causal inferences.

## 6 Conclusion

The key conclusions are as follows: Descriptive analysis shows most residents are satisfied with their residential environment (air/water quality, extreme weather) but concerned about vehicle exhaust hazards; their lifestyle (sleep, leisure, social activities) is generally normal. Baseline regression confirms environmental awareness’ direct positive impact on physical health status: perceptions of extreme weather effects, pesticide/fertilizer hazards, GMO risks, food safety, and fresh produce accessibility significantly boost health, indicating positive environmental cognition benefits health maintenance. Unhealthy lifestyles exert a significant negative impact on health and become a key source of health risks, whereas Chen et al. (29) and Lan et al. (30) did not investigate the quantitative influence of lifestyle choice probability on health. The mediating effect model reveals lifestyle plays an important mediating role: positive environmental perception enhances social fairness, improves sleep quality, and promotes leisure/social participation, ultimately improving physical health. The core contribution of this paper lies in revealing the chain influence mechanism of “environmental perception—lifestyle—physical health,” and the practical significance is reflected in providing accurate data support for government environmental governance and community health intervention.

## Data Availability

The raw data supporting the conclusions of this article will be made available by the authors, without undue reservation.

## Footnotes

1 The data source URL is: http://cgss.ruc.edu.cn/.

2 The environmental perception data are derived from Environmental Module 1 (Page 71) and Environmental Module 2 (Page 78) of the CGSS 2021 questionnaire.

3 The data are sourced from the “Lifestyles” (Page 19) and “Social Attitudes” (Page 22) sections in the “Section A—Core Module” of the CGSS 2021 questionnaire.

4 The data are sourced from the “Health” section (Page 15) in the “Section A—Core Module” of CGSS 2021 questionnaire.

5 The data source URL is: http://cgss.ruc.edu.cn/. The variable data of the models are mainly derived from “Section A—Core Module” and “Environmental Module 1 and 2” in the questionnaire of the Chinese General Social Survey (CGSS2021).

## References

[1] Camanzi L Kaliji S Prosperi P Collewet L Khechen R Michailidis A . Value seeking, health-conscious or sustainability-concerned? Profiling fruit and vegetable consumers in Euro-Mediterranean countries. Br Food J. (2024) 126:303–31. doi: 10.1108/BFJ-12-2023-1151

[2] Guo Y Xie Z. Bridging the gap in one generation: the theory of health social determinants and its international experience. J Peking Univ. (2009) 41:125–8.

[3] Chen Z Zhang C Li J Zhang C. Research on the influence of spatial distribution difference of urban park green space accessibility on population health. Mod Agric Res. (2024) 30:77–86. doi: 10.19704/j.cnki.xdnyyj.2024.12.015

[4] Zhu J Lu C. Air quality, pollution perception, and residents’ health: evidence from China. Toxics. (2023) 11:591. doi: 10.3390/toxics11070591, 37505557 PMC10383338

[5] Hu K Fa X. Rural-urban differences in the relationship between air pollution and health outcomes among middle-aged and older adults: a longitudinal study based on CHARLS and remote sensing data. Sociol Rev China. (2025) 13:29–53.

[6] Jones R Rainey S. Examining linkages between race, environmental concern, health, and justice in a highly polluted community of color. J Black Stud. (2006) 36:473–96. doi: 10.1177/0021934705280411

[7] Rahut D Mishra R Sonobe T Timilsina R. Prevalence of prehypertension and hypertension among the adults in South Asia: a multinomial Logit model. Front Public Health. (2023) 10:1006457. doi: 10.3389/fpubh.2022.1006457, 36777775 PMC9911430

[8] Lin A Lou J Zeng E Li D Zheng L. Impact of energy policies on residential low-carbon behaviors by considering place attachment: evidence from China. Energy Environ. (2025) 36:425–47. doi: 10.1177/0958305X231183683

[9] Lin A Lou J Shan J Li D. How policy satisfaction affects residents' low-carbon behavior: the mediating role of place attachment. J Asian Archit Build Eng. (2025) 24:5672–85. doi: 10.1080/13467581.2024.2412145

[10] Russ P. The potential influences of environmental perception on human health. J Environ Psychol. (1991) 11:1–23. doi: 10.1016/S0272-4944(05)80002-7, 41429700

[11] Adanu S Boakye M Gbedemah S Nyatuame M. Perceptions of environmental and health effects of quarry activities at Klefe in the Ho municipality of the Volta region. GeoHealth. (2025) 9:e2024GH001168. doi: 10.1029/2024GH001168, 40052121 PMC11883180

[12] Huang Q Jin H. Research progress and lessons of influence mechanism of green space on physical activity based on social ecology model. Chin Landscape Archit. (2023) 39:93–8. doi: 10.19775/j.cla.2023.03.0093

[13] Shin M Werner A Strosnider H Hines L Balluz L Yip F. Public perceptions of environmental public health risks in the United States. Int J Environ Res Public Health. (2019) 16:1045. doi: 10.3390/ijerph16061045, 30909505 PMC6466406

[14] Yin J Jiang L Zhang H Zhang J Zhang J Yao N . Combined influence of healthy lifestyles, nutritional and inflammatory status on mortality among US adults with depression. J Psychosom Res. (2025) 193:112131. doi: 10.1016/j.jpsychores.2025.112131, 40286566

[15] Makeen A Gosadi I Jareebi M Muaddi M. Satisfaction-behavior paradox in lifestyle choices: a cross-sectional study of health behaviors and satisfaction levels in Jazan, Saudi Arabia. Healthcare. (2024) 12:1770. doi: 10.3390/healthcare12171770, 39273794 PMC11395601

[16] Subiza M Zabala A Groten D Vozmediano L Sanjuan C Ibarluzea J. Waste-to-energy risk perception typology: health, politics and environmental impacts. J Risk Res. (2023) 26:1101–18. doi: 10.1080/13669877.2023.2259402

[17] Vasekova V. How do people in China perceive water? From health threat perception to environmental policy change. J Environ Stud Sci. (2022) 12:627–45. doi: 10.1007/s13412-022-00773-x

[18] Yazd S Karaman M Fathi S Alsarraf A Alajmi S Rutabian S . Examining the impact of working conditions, lifestyle choices, and demographic factors on mental health of industrial workers. Ment Health Soc Incl. (2024) 28:345–57. doi: 10.1108/MHSI-11-2023-0119

[19] Kalouguina V Wagner J. How do health, care services consumption and lifestyle factors affect the choice of health insurance plans in Switzerland? Risks. (2020) 8:41. doi: 10.3390/risks8020041, 41291439

[20] Timmons S Papadopoulos A Lunn P. Survey instructions bias perceptions of environmental health risks. J Risk Res. (2024) 27:932–50. doi: 10.1080/13669877.2024.2421006

[21] Sprague N Zonnevylle H Hall L Williams R Dains H Liang D . Environmental health perceptions of urban youth from low-income communities: a qualitative photovoice study and framework. Health Expect. (2023) 26:1832–42. doi: 10.1111/hex.13776, 37317064 PMC10485307

[22] Janmaimool P Chontanawat J Chudech S. The effects of perceptions of environmental health risk and environmental risk on sustainable infectious waste management behaviours among citizens in Bangkok, Thailand. Clean Responsible Consum. (2024) 12:100175. doi: 10.1016/j.clrc.2024.100175

[23] Rashidi T Auld J Mohammadian A. A behavioral housing search model: two-stage hazard-based and multinomial Logit approach to choice-set formation and location selection. Transp Res A. (2012) 46:1097–107. doi: 10.1016/j.tra.2012.01.007

[24] Caudill S. Pooling choices or categories in multinomial Logit models. Stat Pap. (2000) 41:353–8. doi: 10.1007/BF02925928

[25] Ding H Su Z Liu X. A modified multinomial baseline Logit model with Logit functions having different covariates. Commun Stat Simul Comput. (2020) 49:2861–75. doi: 10.1080/03610918.2018.1529238

[26] Shivam K Mahmudur R Meghan W. How daily activities and built environment affect health? A latent segmentation-based random parameter Logit modeling approach. Travel Behav Soc. (2023) 33:100624. doi: 10.1016/j.tbs.2023.100624, 41429700

[27] Liu J Yin C Sun B. Associations between neighborhood environments and health status among Chinese older people during the pandemic: exploring mediation effects of physical activity. J Transp Health. (2024) 35:101757. doi: 10.1016/j.jth.2024.101757

[28] Zeng X Yang G. Influencing factors of air-quality perception in China: What is constructed? What is hidden? Front Environ Sci. (2023) 10:1088895. doi: 10.3389/fenvs.2022.1088895, 41427048

[29] Chen Y Zhang Z Shi P Song X Wang P Wei X . Public perception and responses to environmental pollution and health risks: evaluation and implication from a national survey in China. J Risk Res. (2017) 20:347–65. doi: 10.1080/13669877.2015.1057199

[30] Lan G Yuan Z Maddock J Cook A Chu Y Pan B . Public perception of air pollution and health effects in Nanchang, China. Air Qual Atmos Health. (2016) 9:951–9. doi: 10.1007/s11869-016-0397-0

